# Evaluation of a Post-Operative Rehabilitation Program in Patients Undergoing Laparoscopic Colorectal Cancer Surgery: A Pilot Study

**DOI:** 10.3390/ijerph18115632

**Published:** 2021-05-25

**Authors:** Sveva Maria Nusca, Attilio Parisi, Paolo Mercantini, Marcello Gasparrini, Francesco Antonio Pitasi, Alessandra Lacopo, Vincenzo Colonna, Giulia Stella, Claudia Cerulli, Elisa Grazioli, Eliana Tranchita, Flavia Santoboni, Eleonora Latini, Donatella Trischitta, Mario Vetrano, Vincenzo Visco, Antonio Pavan, Maria Chiara Vulpiani

**Affiliations:** 1 PhD Course in “Translational Medicine and Oncology”, Department of Medical and Surgical Sciences and Translational Medicine, Sant’Andrea University Hospital, “Sapienza” University of Rome, 00189 Rome, Italy; 2 Department of Movement, Human and Health Sciences, University of Rome “Foro Italico”, 00135 Rome, Italy; attilio.parisi@uniroma4.it (A.P.); claudia.cerulli@uniroma4.it (C.C.); elisagrazioliphd@gmail.com (E.G.); eliana.tranchita@gmail.com (E.T.); 3 Department of Medical and Surgical Sciences and Translational Medicine, Sant’Andrea University Hospital, “Sapienza” University of Rome, 00189 Rome, Italy; paolo.mercantini@uniroma1.it; 4 Department of General Surgery, Sant’Andrea University Hospital, “Sapienza” University of Rome, 00189 Rome, Italy; marcellogasparrini1@gmail.com; 5 Physical Medicine and Rehabilitation Unit, Department of Medical and Surgical Sciences and Translational Medicine, Sant’Andrea University Hospital, “Sapienza” University of Rome, 00189 Rome, Italy; francescopitasi@hotmail.it (F.A.P.); lacopo.alessandra90@gmail.com (A.L.); vincenzo.colonna@hotmail.it (V.C.); giuliastella18@gmail.com (G.S.); flavia.santoboni@gmail.com (F.S.); eleonora_gib@yahoo.it (E.L.); donatella.trischitta@uniroma1.it (D.T.); mario.vetrano@uniroma1.it (M.V.); mariachiara.vulpiani@uniroma1.it (M.C.V.); 6 Department of Clinical and Molecular Medicine, Sant’Andrea University Hospital, “Sapienza” University of Rome, 00189 Rome, Italy; vincenzo.visco1@uniroma1.it (V.V.); antonio.pavan@uniroma1.it (A.P.)

**Keywords:** oncology, physical exercise, combined exercise, supervised exercise, Enhanced Recovery after Surgery (ERAS), quality of life

## Abstract

This pilot study explores the effects of a post-operative physical exercise program on the quality of life (QoL) and functional and nutritional parameters of patients that underwent laparoscopic colorectal cancer surgery, compared to usual care alone. The intervention group (IG) attended a 2-month-long supervised and combined exercise–training program during the post-operative period. Both IG and control group (CG) participated in the QoL, functional, and nutritional assessments before exercise training (T0), 2 months after the beginning of the exercise (end of treatment) (T1), and 2 (T2) and 4 (T3) months from the end of treatment. Eleven patients with colorectal cancer that underwent laparoscopic surgery were enrolled (six intervention; five control). The IG showed significant improvements compared to the CG in “Physical functioning” (PF2) (*p* = 0.030), “Cognitive functioning” (CF) (*p* = 0.018), and “Fatigue” (FA) (*p* = 0.017) of the European Organization for Research and Treatment of Cancer Quality of Life-C30 Questionnaire (EORTC QLQ-C30) at T1; in SMWT (*p* = 0.022) at T1; in PF2 (*p* = 0.018) and FA (*p* = 0.045) of EORTC QLQ-C30 at T2, in phase angle (PhA) of bioelectrical impedance analysis (*p* = 0.022) at T3. This pilot study shows that a post-operative, combined, and supervised physical exercise program may have positive effects in improving the QoL, functional capacity, and nutritional status in patients that undergo laparoscopic colorectal cancer surgery.

## 1. Introduction

Cancer and its treatments often produce significant morbidities that undermine the quality of life (QoL) in survivors [1]. Oncological rehabilitation aims to improve the QoL of patients with cancer, helping them to adapt to living standards as close as possible to what they were used to before the disease. Previous studies have shown that exercise training and dance programs have a positive effect on cardiorespiratory fitness, hand grip strength, quality of life, and psychological aspects in oncological patients [2,3,4,5]. A meta-analysis of randomized controlled trials (RCTs) reported beneficial effects of physical exercise during and after anti-tumor treatment [6]. Colorectal cancer is one of the most common neoplasms worldwide, being the third most common cancer in men and second in women [7]. In Italy, there were over 27,000 and 22,000 new diagnoses of colorectal cancer in 2019, in men and women respectively. In recent years, the 5-year colorectal cancer survival rate has increased due to medical and surgical therapeutic advances and improvement of screening programs. Currently, the 5-year survival is 66% [8]. Patients undergoing colorectal surgery often experience a reduction in physiological and functional capacity even in absence of complications leading to a reduction in QoL [9]. Early postoperative mobilization is strongly recommended, as part of the Enhanced Recovery after Surgery (ERAS) guidelines following colorectal surgery [10]. Nonetheless, physical activity interventions are not part of standard practice following surgical treatment for colorectal cancer. A recent Cochrane review on the impact of physical activity intervention in people with non-advanced colorectal cancer (staged as T1-4 N0-2 M0) found evidence of positive effects of exercise interventions on aerobic fitness, cancer-related fatigue, and health-related QoL at immediate-term and short-term follow-up [11]. Most of the included studies investigated the effect of exercise offered several weeks or months after the end of active treatment (time beyond treatment ranging between 2 months and 5 years in 10 out of 16 studies included). The literature concerning the role of physical exercise after surgery is less consistent [12,13,14,15]. To our knowledge, the literature lacks studies investigating the effect of an early, combined, and supervised physical exercise program on QoL in patients that have undergone surgical treatment for colorectal cancer. This pilot study explores the effects of a post-operative combined and supervised physical exercise training on the quality of life and functional and nutritional parameters of patients that underwent laparoscopic colorectal cancer surgery, compared to usual care alone. The feasibility and safety of the study were also considered.

### 1.1. The Primary Objective of the Study

The primary objective of this study is to explore the effects of a 2-month-long post-operative combined and supervised exercise training (ET) in the quality of life of patients that underwent laparoscopic colorectal surgery after the exercise program (T1) was completed, compared to usual care alone.

### 1.2. Secondary Objectives of the Study

To investigate whether any differences in quality of life observed at T1 are maintained in the longer term, in particular at 2 (T2) and 4 (T3) months from the end of the exercise program.

To explore sleep quality, anxiety and depression, functional capacity, physical performance, muscle strength, Body Mass Index (BMI), muscle mass, and nutritional status between intervention and control group at T1, T2, and T3.

To explore all clinical outcomes within the intervention group and control group from baseline (T0) to the end of the exercise program (T1) and at 2 (T2) and 4 (T3) months from the end of the intervention.

## 2. Materials & Methods

### 2.1. Study Design

This is a pilot study wich preliminary results will be confirmed in a Randomized Controlled Trial (RCT) approved by the Institutional Review Board of the “Sapienza” University of Rome (RS 5304/2019) and conducted according to good clinical practice and the ethics of the Helsinki declaration [16].

### 2.2. Eligibility Criteria and Enrollment

The recruitment took place from May 2019 to July 2020 at the Surgical Emergency Unit and the Week-Surgery Unit of Sant’Andrea Hospital, Rome, Italy, by a physiatrist of the Physical Medicine and Rehabilitation Unit of the same hospital. Patients with newly diagnosed colorectal cancer that underwent laparoscopic resective surgery with curative intent were eligible.

Inclusion criteria were as follows:

- age 40–80;
- histologically confirmed diagnosis of primary colon or rectal neoplasm;
- physically inactive patients (duration of physical activity <150 min per week);
- Karnofsky Perfomance Status (KPS) > 60 and able to walk ≥ 60 m. The Karnosky Performance Status is one of the most used validated scales to define the functional status of the cancer patient. A KPS ≤ 60 indicates the inability to work and severe difficulty in carrying out activities of daily living and personal care of the cancer patient [17].

Exclusion criteria were as follows:

- laparotomic surgery;
- pregnancy;
- relapsing cancer or metastasic cancer;
- simultaneous diagnosis of other neoplasms;
- cancer treatment in the 5 years before recruitment;
- severe cardiovascular, pulmonary, orthopedic, neurological pathologies;
- cognitive impairment;
- regular use of immunosuppressive drugs.

**Figure 1:**
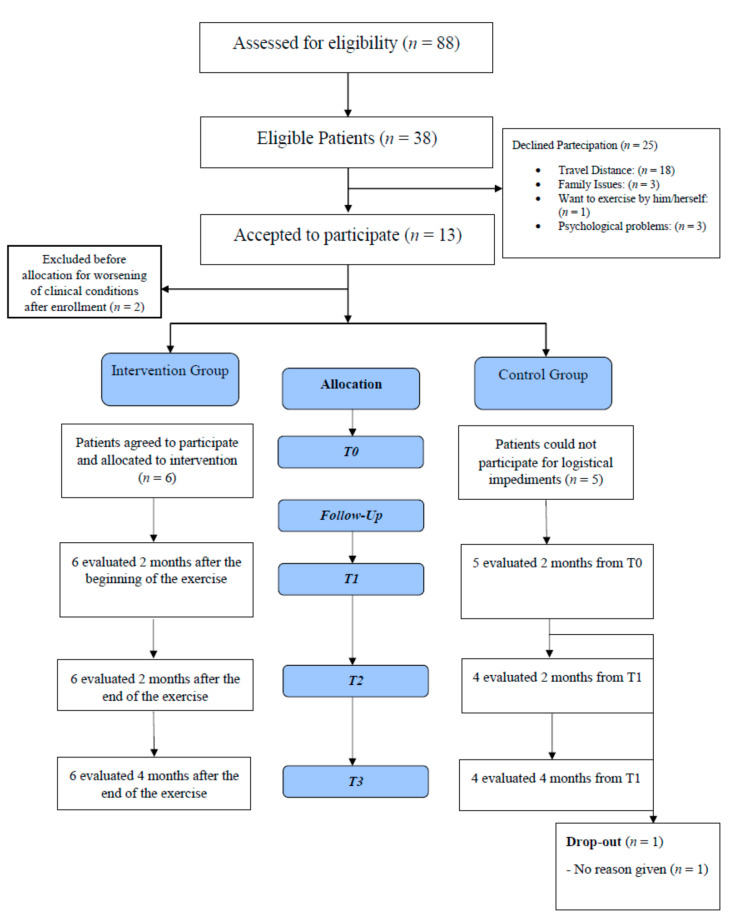
Consort flow diagram.

During the assessment, the study was explained in detail, and the inclusion and exclusion criteria were evaluated. Patients who declined their participation were asked about the motivation, and their answers were recorded. Eligible patients who agreed to participate signed written informed consent. A surgical consultation was carried out concurrently with the first post-surgical follow-up visit approximately ten days after the intervention, to assess eligibility for physical activity. Then, a cardiological screening of patients was carried out by a Sports Medicine Specialist at “Foro Italico” University of Rome, Italy, to determine eligibility for physical exercise (PE) program. The proposed participant flow through the study is shown in Figure 1.

After completing the baseline data, eligible patients were assigned to the intervention group (IG). All the patients eligible for exercise but not participating in the exercise training program because of logistical reasons were considered as the control group (CG). The outcome measures were administered before exercise training (T0), 2 months after the beginning of the exercise (end of treatment) (T1), and 2 (T2) and 4 (T3) months from the end of treatment, respectively. The assessments at T0, T1, T2, and T3 were carried out by the same physiatrist.

### 2.3. Intervention

Patients in IG underwent a post-operative (approximately 15–20 days after the intervention) PE program of the duration of 2 months. PE was a supervised, moderate-intensity aerobic type training during the first month after surgery, and combined training (moderate-intensity aerobic exercise, and muscle strengthening) during the second month after surgery.

#### 2.3.1. Physical Exercise

The exercise program was designed following the guidelines of the American College of Sports Medicine [18] and the Canadian Society for Exercise Physiology [19]. According to the guidelines, a volume of weekly activity of 150 min of moderate-intensity aerobic exercise per week, and two to three weekly sessions of muscle strength training, are recommended. Both combined exercise and aerobic exercise alone were safe and effective in various cancer studies [20,21,22].

Each patient underwent three weekly sessions of physical exercise, lasting 1 h. Each session was supervised by a Sport Sciences specialized trained to the protocol and by a physiatrist. Each session included a warm-up phase (10 min); aerobic exercise (40 min) in the first month of the post-surgical period, and a combination of aerobic (20 min) and muscle-strengthening exercise (20 min) in the second month; and then a cool-down phase (10 min). Before and at the end of each exercise session, the physiatrist assessed blood pressure, heart rate, and blood oxygen saturation and asked patients to report any immediate symptoms.

The exercise program was suspended if one of these conditions occurred: cardiovascular, pulmonary, traumatic events, hemoglobin ≤10 g/dL until values are restored > 10 g/dL, neutropenia (absolute neutrophil count less than 0.5 × 109 µL), thrombocytopenia (platelet count less than 50 × 109 µL), sudden onset of nausea and vomiting within 24–36 h of exercise, unusual fatigue or decreased muscle strength, disorientation, decreased vision, pain, peripheral neuropathies with the reduction in muscle strength, ataxia, and loss of balance. The exercise program was resumed when the aforementioned conditions were resolved.

#### 2.3.2. Aerobic Exercise

Aerobic exercises were performed on a treadmill and stationary bike, with gradually increasing intensity between 60 and 70% of the patient’s maximum heart rate determined by the Karvonen formulae [23]. The intensity was adjusted according to the results obtained from the Borg scale of perceived exertion. The Borg Scale aims to evaluate the subjective perception of physical effort during physical activity [24]. Patients wore heart rate monitors to ensure they were training at the pre-set level of intensity.

#### 2.3.3. Muscle-Strengthening Exercise

Muscle strengthening exercises were performed at 30–50% of predicted 1-RM values estimated from the Brzycki formula [25] using a leg press for the lower limb and the Cable Station–Ercolina Rehab for the upper arm. Every two weeks, the load was increased when the patient perceived it as very light (Borg value CR10 = 1). Two sets of ten repetitions for each exercise were performed.

#### 2.3.4. Control Group

Patients of the control group were encouraged to maintain a physically active lifestyle (as walking and taking the stairs instead of using an elevator).

### 2.4. Outcome Measures

#### 2.4.1. Primary Outcome

EORTC QLQ-C30 scale: Quality of life was assessed by the Italian version of the EORTC QLQ-C30 scale (European Organization for Research and Treatment of Cancer Quality of Life-C30 questionnaire), which is specific for neoplastic patients. The EORTC QLQ-C30 is a validated and reliable score, consisting of a two-item global health and QoL scale, five multi-item functional scales (Physical, Role, Cognitive, Emotional, and Social), three multi-item symptom scales (Fatigue, Pain, and Nausea/Vomiting), a global health/quality of life scale, five single-item symptom scales (Dyspnea, Insomnia, Loss of Appetite, Constipation, and Diarrhea) and a single-item financial impact scale. A higher score on the Global Health Scale indicates a better quality of life. In functional subscales, a higher score corresponds to a better level of function. In the symptom subscales, a higher score indicates worse symptoms [26].

#### 2.4.2. Secondary Outcomes

The Six Minutes Walking Test (6MWT): The functional capacity of the patient was assessed by the Six Minutes Walking Test (6MWT). This test is a validated measure of post-surgical recovery, representing a measurement of the physical effort required by the activities of daily life [27,28,29]. It’s a global assessment of the subject’s ability to cope with the functional requirements during physical exercise and integrates different components of functional capacity (i.e., balance, speed, and endurance). Patients performed the 6MWT walking back and forth for 6 min in a space of 20 m, at a brisk pace, according to current guidelines. The subjects began the test after a resting period, and they could rest during the test if necessary [30].

Short Physical Performance Battery (SPPB): The physical performance was evaluated through the Short Physical Performance Battery (SPPB). The SPPB scale is a short battery of tests evaluating the functionality of the lower limbs. This battery consists of 3 different sections. The first section consists of evaluating the patient’s balance while maintaining 3 different positions:

- position with feet together for 10;
- semi-tandem position for 10 s (big toe on the side of the heel);
- tandem position for 10 s (big toe behind the heel).

The score in this section varies from 0 if the patient is unable to maintain the position with feet together for at least 10 s, to 4 if he can complete all three tests. The second test is aimed to evaluate the gait speed on 4 linear meters. The section score varies from 0 if unable to perform the test, to 1 point if the performance lasts longer than 8.7 s, to a maximum of 4 points if the patient manages to complete the task in less than 4.8s. The third section investigates the ability and the time taken to perform the sit-to-stand from a chair or 5 consecutive times without using the upper limbs. The score varies from 0 if unable to carry out the test or the performance has a duration greater than 60 s, to a maximum of 4 if this test is carried out in less than 11.2 s. The total score on the scale ranges from 0 to 12 [31].

- Handgrip Strength test: Muscle strength was assessed by the Handgrip Strength test. The measure of handgrip strength is evaluated using a handheld dynamometer, equipped with a spring set of 20 kg capacity. The participants were seated in a chair without armrests with the elbow flexed in a 90° angle position and were asked to squeeze the handgrip instrument three times shortly maximally with one-minute rest between measurements. Once the grip width was adjusted, the subject held the dynamometer while the operator encouraged him or her to tighten with the maximum possible force. The value was then recorded. The final value of grip strength is the arithmetical mean of the three values. The measure of handgrip strength is used in numerous studies, conducted in different populations. Upper limb strength is strictly correlated with lower extremities muscle strength and calf sectional area [32,33,34,35,36]. Moreover, baseline handgrip strength has a linear relationship with disability in ADL [37].
- Skeletal Muscle Mass Index (SMI): muscle mass was estimated by calculating the Skeletal Muscle Mass Index (SMI) using a bioimpedance meter. According to the European Society for Clinical Nutrition and Metabolism (ESPEN), bioelectrical impedance analysis (BIA) is considered a quick, easy to perform, and non-invasive method to estimate body composition [38]. BIA is currently considered a widespread and validated body composition assessment technique in various clinical contexts [39,40]. The muscle mass index (SMI) is calculated as the ratio of skeletal muscle mass (SM) and the square of height (h2) (SM/h2). The European Working Group on Sarcopenia in Older People (EWGSOP) [41] defines BIA as a “good portable alternative” method; due to its affordability, portability, and ease of execution, it is recommended in the systematic and repeated assessment of muscle mass in clinical practice.
- The Phase Angle (PhA): nutritional status is assessed by calculating the Phase Angle (PhA). It is a value obtained from the reactance (Xc) and the resistance (R) parameters, assessed during BIA. PhA is calculated using the formula: arc tan (Xc/R) × (180/π). PhA is considered a valuable indicator of cellular health and integrity [42]. Thus, PhA is considered a prognostic marker in several clinical conditions, including cancer, as it represents either cell death or malnutrition, which are characterized by changes in cellular membrane integrity [43,44].
- The Italian version of the Hospital Anxiety and Depression Scale (HADS): Anxiety and depression were assessed using the Italian version of the Hospital Anxiety and Depression Scale (HADS) [45]. The questionnaire consists of 14 items, with each score ranging from 0 to 21. A value higher than 8 suggests the presence of a mood disorder [46].
- The Pittsburgh Sleep Quality Index (PSQI): Sleep quality was evaluated using the Pittsburgh Sleep Quality Index (PSQI). It is a validated, self-administered questionnaire that consists of 19 items that assess the quality of sleep for 1 month [47,48].
- Post-operative complications: The surgical complications were recorded. They were classified according to their severity using the Dindo–Clavien Classification. Grade I indicates a complication that requires management in the patient’s bed; grade II a drug treatment; grade III a radiological, endoscopic, or surgical treatment; and grade IV intensive care [49].

## 3. Statistical Methods

### Statistical Analysis

The descriptive statistics included the median with range for quantitative variables and percentage and tables of frequencies for qualitative variables. A nonparametric approach was considered, based on the low number of patients. The Mann–Whitney test was performed to compare the intervention group versus the control group at the four times (T0, T1, T2, T3). The significance of the change in each group at all follow-up times was determined by nonparametric Wilcoxon signed-rank test. The analysis was planned according to the intention-to-treat principle. All tests were two-tailed with a level of significance of *p* < 0.05. IBM SPSS Statistics ver. 20.0 (Chicago, IL, USA) was used for the statistical analyses.

The feasibility of the study was assessed through the eligibility rate, the enrollment rate, and the exercise adherence rate. The eligibility rate was assessed by the number of eligible patients divided by the total number of elements on the sampling frame. The enrollment rate was assessed by the number of patients included in the study divided by the total number of eligible patients. The exercise adherence rate was assessed by the number of exercise sessions attended out of the 24 sessions scheduled for each patient.

Safety was assessed by monitoring any serious adverse events that occurred during the ET period.

## 4. Results

Between May 2019 and July 2020, 88 patients with colorectal cancer who had undergone resection surgery were evaluated at the Surgical Emergency Unit and the Week-Surgery Unit of Sant’Andrea Hospital. Of the 38 eligible patients, 13 agreed to participate, while 25 refused. Of the 13 who wanted to participate, 5 could not attend for logistical impediments and became part of the control group, while 2 patients were excluded after enrollment due to worsening of their physical condition. In sum, 6 patients were enrolled in the intervention group (IG) and 5 patients in the control group (CG). No significant intergroup differences (*p* > 0.05) were found at baseline assessment. In the intervention group, 6 patients were evaluated at T1, T2, and T3. In the control group, 5 patients were evaluated at T1, and 4 patients were evaluated at T2 and T3 because 1 patient dropped out between T1 and T2. Patients in the control group reported that they did not follow a physically active lifestyle.

The Consort Flow Diagram is shown in Figure 1.

### 4.1. Reasons for the Refusal of Exercise Training

The self-reported reasons for the refusal to participate in the study were: 18 patients were not willing to travel distance, 3 patients for family issues, 1 patient wanted to exercise by him/herself; 3 patients were not willing to participate due to their psychological status (Figure 1).

### 4.2. Demographic, Clinicopathologic, and Operative Variables

The median age of the intervention group was 63.5 years (43.0–80.0); 83.3% was male and 16.7% female. The median age of the control group was 73.0 years (51.0–80.0); 33.3% was male and 66.7% female. The median BMI of the IG was 21.4 (17.2–25.7); the median BMI of the CG was 24.0 (22.0–41.4). The median Karnofsky Performance Status (KPS) was 80.0 (70.0–80.0) for both the intervention group and the control group. 83.3% of the IG underwent laparoscopic colon resection; 16.7% of IG underwent laparoscopic rectal resection. 66.7% of CG underwent laparoscopic colon resection; 33.3% of CG underwent laparoscopic rectal resection. No patient undergoing rectal resection had a new stoma. Tumors were staged according to the current American Joint Commission on Cancer/International Union against Cancer Tumor-Node-Metastasis (TNM)staging [50]. All patients in both the intervention and control group started chemotherapy around the second month of the exercise program.

Patients’ demographic, clinicopathologic, operative characteristics, and primary outcome EORTC QLQ-C30 at baseline are summarized in Table 1.

### 4.3. Clinical Results

#### 4.3.1. Primary Objective

At the evaluation times between groups for the Mann–Whitney U test, a statistically significant difference in the following subscales of the EORTC QLQ-C30 scale was found: “Physical functioning” (PF2) (*p* = 0.030), “Cognitive functioning” (CF) (*p* = 0.018), and “Fatigue” (FA) (*p* = 0.017) at T1 in favor of the intervention group (IG) (Table 2).

#### 4.3.2. Secondary Objectives

At the evaluation times between groups for the Mann–Whitney U test, a statistically significant difference was maintained in the following subscales of the EORTC QLQ-C30 scale: “Physical functioning” (PF2) (*p* = 0.018) and “Fatigue” (FA) (*p* = 0.045) at T2 in favor of the intervention group (IG) (Table 3). No statistically significant differences were found at T3 (Table 4). No significant differences between IG and CG were observed in PSQI and HADS anxiety and depression at T1, T2, and T3 (Table 5). Among the functional parameters, a statistically significant difference in 6 Minute Walking Test (SMWT) at T1 (*p* = 0.022) in favor of the IG was found. (Table 5). Among the nutritional parameters, a statistically significant difference in PhA-BIA at T3 (*p* = 0.022) in favor of the IG was found. (Table 5).

At Wilcoxon signed-rank test in the intervention group, it was observed a statistically significant difference in the EORTC QLQ-C30 subscale of “Social Functioning” at T0 vs. T3 (*p* = 0.027) and in Fatigue at T0 vs. T3 (*p* = 0.046). Among the functional parameters, it was observed a statistically significant difference in 6MWT at T0 vs. T1 (*p* = 0.028). Among the nutritional parameters, it was observed a statistically significant difference in PhA-BIA at T0 vs. T2 (*p* = 0.028) and T0 vs. T3 (*p* = 0.027) (Table 6).

At Wilcoxon signed-rank test in the control group, no statistically significant difference was observed in any of the parameters at each follow-up (Table 7).

### 4.4. Feasibility

The eligibility and the enrollment rates were 43% and 29% respectively. The exercise adherence rate for the IG was 100%.

### 4.5. Safety

No adverse effects were observed in the intervention group during the exercise training.

### 4.6. Post-Surgical Complications

No post-surgical complications were observed in both groups at each follow-up.

## 5. Discussion

In the present pilot study, the intervention group showed significant improvements compared to the control group in “Physical functioning”, “Cognitive functioning”, and “Fatigue” of EORTC QLQ-C30 at T1; in the Six Minutes Walking Test (6MWT) at T1; in “Physical functioning” and “Fatigue” of EORTC QLQ-C30 at T2, in phase angle of bioelectrical impedance analysis at T3.

In particular, we observed that a 2-month-long post-operative combined and supervised exercise-training program improved QoL in patients that underwent laparoscopic resective surgery for colorectal cancer compared to the control group. A consistent number of trials reported beneficial effects of physical exercise in patients treated for colorectal cancer, but the interventions are often administered during and/or after adjuvant treatment, many weeks or months after surgery [11]. Literature addressing the role of the rehabilitation after surgery is less consistent and the optimal exercise during the early survivorship stage of colorectal cancer is still unknown [51].

QoL is a subjective multi-dimensional concept encompassing physical, psychological, and social domains [52,53]. QoL provides clinicians with important information to consider alongside biological indicators for prognostic and management judgments [54]. Our results agree with the current literature. A Cochrane review on 16 RCT, involving colorectal cancer patients, exclusively shows that physical exercise is effective in improving health-related quality of life. However, the largest part of the studies included in the review involves physical exercise provided during or after chemotherapy [11].

In our study, exploratory non-parametric analyses suggested that the intervention group had a significant improvement compared to the control group, at the end of the physical intervention (T1) and 2 months from the end of the intervention (T2) in the physical function item (PF2) and fatigue (FA) of EORTC QLQ C30; at T1 in the cognitive function item (CF) of EORTC QLQ C30. Physical exercise has clinically important effects on physical functioning in cancer patients, as shown by a metanalyis [55]. However, most included studies involve breast cancer patients or mixed cancer populations. Our study explored the positive effect of exercise training on physical functioning in a sample of exclusively non-advanced, colorectal cancer patients after surgery. Our study also showed a promising role of physical intervention in improving cognitive function, despite further research being needed [56]. The “cognitive functioning” subscale of EORTC QLQ 30 is not a specific tool to assess cognitive outcomes, nonetheless it is used in some studies in cancer patients [57,58]. A recent prospective longitudinal study evaluating cognitive function in patients with colorectal cancer showed that 43% had objective cognitive impairment shortly after diagnosis, compared with 15% in healthy controls without cancer [59]. Our study suggested that the exercise significantly reduced fatigue in the post-operative period and 2 months after the end of the exercise program. Cancer-related fatigue affects between 60% to 96% of people with cancer during and following chemotherapy, radiotherapy, or surgery. It negatively affects mood and quality of life [60] and it can interfere with one’s ability to carry out daily activities [61]. Our results agree with the current literature. A review [62]^.^ demonstrated that supervised multi-modal exercise improves cancer-related fatigue compared with conventional care. As demonstrated by a recent Cochrane review [11], exercise is an effective treatment in treating cancer-related fatigue during and following treatment for non-advanced colorectal cancer. Through improved cardiorespiratory fitness (CRF) and muscle strength, physical activity may help manage cancer-related fatigue. In our study, we observed a statistically significant improvement at T1 of the 6-min walk test for distance (6MWT) in the intervention group compared to the control group. 6MWT is a common method to estimate CRF in clinical practice even if it may not necessarily provide an accurate estimation of CRF as the cardiopulmonary exercise testing [63]. 6MWT measures the functional capacity of patients and it is a validated measure of post-surgical recovery after colon resection surgery [28,29]. In the study of Awasthi et al. [14], supervised multimodal prehabilitation and rehabilitation exercise training improves functional capacity in patients undergoing colorectal surgery. Gillis et al. [15] achieved improvements in functional capacity in a patient undergoing both prehabilitation and rehabilitation physical exercise, or rehabilitation alone. A recent pilot RCT by Mascherini et al. [12] showed the promising, preliminary results of a mixed exercise approach using 1 month of supervised resistance exercise and unsupervised home-based aerobic exercise, followed by unsupervised home-based aerobic exercise alone on non-metastatic colorectal adenocarcinoma, in providing faster recovery after surgery. Our data also showed a statistically significant difference in phase angle between the intervention group and control group at T3 in favor of the IG. The phase angle is an expression of mass and quality of soft tissues, and cellular health [42]. In colorectal cancer patients, an increase in phase angle was associated with an increase in physical and role function scales and a decrease in fatigue of the EORTC questionnaire, indicating improved functional aspects of quality of life [44].

Consistently with these findings, the within-groups analyses showed that in the intervention group fatigue is significantly reduced from T0 to T3, 6mwt significantly improved from T0 to T1, and PhA significantly improved from T0 to T2 and from T0 to T3. The EORTC QLQ “social functioning” subscale also significantly improved from T0 a T3. Other studies detected an improvement in social functioning in cancer patients after physical exercise intervention [64]. The improvement could be ascribed to the psychosocial aspects of supervised exercise bringing social interactions, and the level of support during exercise. No significant differences were found in the control group. Our pilot study has several limitations: the small sample size, the lack of randomization, the lack of the assessor blinding. In this pilot study, eligible patients were assigned to the intervention group. All the patients eligible for exercise but not participating in the exercise training program because of logistical reasons were considered as the control group. So the lack of randomization could have limited the clinical impact of our conclusions. However, this study should be considered a proof-of-principle that post-operative rehabilitation program in patients undergoing laparoscopic colorectal cancer may have positive effects in improving the QoL, functional capacity, and nutritional status. A future randomized and controlled study will be done to confirm our results.

The main limitation of the study was the small sample size. Enrollment was more difficult than anticipated: from a total of 88 patients with a colon cancer diagnosis that were brought to our attention, only 38 patients were initially identified as potentially eligible. Among them, only 13 patients agreed to take part in the study and were enrolled. Two were excluded before allocation for worsening of their clinical conditions. The enrollment rate is 29%, lower than in previous physical activity studies in colorectal cancer (CRC) (37–41%) [22,64,65], prostate cancer (37%) [66], and higher than in a breast cancer study (19%) [67]. The absolute number of colorectal cancer participants recruited was lower than expected. The most frequent motivation recorded is “distance” (66.7%). Logistical barriers could be one of the main hurdles that prevent participation in an on-site ET program [68]. To improve enrollment rates, it will be important to adopt a counseling approach, which was already validated in other health conditions [69]. Future studies could be multicenter and could offer incentives for participation or transportation services.

Italian national “lockdown” enforced by the Italian Government during COVID-19 pandemics also played a role, hampering enrollment and hindering the availability of the rehabilitation structure. Although the enrollment rate was low, there was excellent adherence to the supervised exercise program. The patients enrolled were highly motivated in attending the exercise sessions. Moreover, no adverse effects were observed in the intervention group during the exercise training. The statistical analyses in this context had only an explorative role, due the small sample size and possible selection bias. The non-parametric tests were considered because they are the method of choice for small patients’ group. Although our preliminary results should be interpreted with caution due to the small sample size, the present study has the following strengths: (1) the results suggest a positive effect of early post-operative exercise in patients that underwent surgery for colorectal cancer; (2) we opted for supervised exercise, which produces better outcomes compared to unsupervised exercise as demonstrated by literature [70,71,72]; (3) our PE program followed the American College of Sports Medicine [18] and the Canadian Society for Exercise Physiology [19] recommendations. Future studies could adopt a presurgical baseline evaluation, to further investigating the relationship between surgery and deterioration of functional status and quality of life and a perioperative design, despite enrollment in such kind of studies appears to be challenging, as described in previous studies [73,74]. “Peri-rehabilitation” is a novel field in oncologic rehabilitation [75,76]. It consists of a process of pre and post-surgical optimization [64], enhancing the individual’s functional capacity and improving their tolerance to upcoming stressors [77].

Based on our data, the present study suggests that supervised and combined physical exercise may have a role in the management of the post-operative phase in patients treated for colorectal cancer. The results should be considered preliminary due to the small sample size and the lack of randomization. The early post-operative phase may potentially be a window of opportunity to help survivors recovering and preparing them for further treatment if it is needed.

## 6. Conclusions

The present pilot study shows that post-operative rehabilitation may have a role in the management of the post-surgical phase in colon cancer patients. Our results will be confirmed by an RCT with a larger sample of patients.

## Figures and Tables

**Table 1:** Patients’ demographic, clinicopathologic, and operative characteristics at baseline.

Variables	IG (*n* = 6)	CG (*n* = 5)	*p*-Value
Age-(median, range)	63.5 (43.0–80.0)	73.0 (51.0–80.0)	0.580
Sex-n. (%)			
Male	5 (83.3)	2(33.3)	
Female	1 (16.7)	3 (66.7)	
BMI-(median, range)	21.4 (17.2–25.7)	24.0 (22.0–41.4)	0.200
KPS (median, range)	80.0 (70.0–80.0)	80.0 (70.0–80.0)	0.540
Neoplasm type-n. (%)			
Colon cancer	5 (83.3)	3 (66.7)	
Rectal cancer	1 (16.7)	2 (33.3)	
TNM Cancer stage-n. (%)			
Stage IIA	1 (16.7)	1 (20.0)	
Stage IIB	2 (33.3)	1 (20.0)	
Stage IIIB	3 (50.0)	3 (60.0)	
Type of resection-n. (%)			
Colon *	5 (83.3)	3 (66.7)	
Rectum **	1 (16.7)	2 (33.3)	
New Stoma-n. (%)	0 (0.0)	0 (0.0)	
Chemotherapy-n. (%)	6 (100.0)	5 (100.0)	
EORTC QLQ-C30-(median, range)			
QL2	50.0 (16.7–83.3)	58.0 (50.0–83.3)	0.910
PF2	93 (60.0–100.0)	90.0 (87.0–93.0)	0.733
RF2	66.5 (33.0–100.0)	67.0 (50.0–100.0)	0.692
EF	79.5 (42.0–93.0)	75.0 (57.0–83.0)	0.580
CF	100.0 (83.0–100.0)	83.3 (83.0–84.0)	0.442
SF	67.0 (33.0–83.0)	84.0 (66.6–100.0)	0.443
FA	38.8 (0.0–66.7)	33.3 (22.0–53.3)	0.854
NV	0.0 (0.0–50.0)	0.0 (0.0–16.7)	0.560
PA	16.7 (0.0–66.7)	0.0 (0.0–33.3)	0.561
DY	0.0 (0.0–33.3)	0.0 (0.0–33.3)	0.892
SL	33.3 (0.0–66.7)	0.0 (0.0–33.3)	0.609
AP	33.4 (0.0–100.0)	33.3 (0.0–66.6)	0.564
CO	16.7 (0.0–66.7)	0.0 (0.0–33.3)	0.242
DI	16.7 (0.0–33.3)	0.0 (0.0–33.3)	0.326
FI	0.0 (0.0–33.3)	0.0 (0.0–33.3)	0.892

Abbreviations: IG, Intervention Group; CG, Control Group; BMI, Body Mass Index; KPS: Karnofsky Performance Status; TNM = tumor–node–metastasis; * Includes right and left hemicolectomy and sigmoid resection. ** Includes anterior resection. EORTC QLQ-C30, European Organization for Research and Treatment of Cancer Quality of Life-C30 Questionnaire; QL, Global Health Status/Quality of Life; PF, Physical Functioning; RF, Role Functioning; EF, Emotional Functioning; CF, Cognitive Functioning; SF, Social Functioning; FA, Fatigue; NV, Nausea and Vomiting; PA, Pain; DY, Dyspnea; SL, Insomnia; AP, Appetite Loss; CO, Constipation; DI, Diarrhea; FI, Financial Difficulties; PSQI, Pittsburgh Sleep Quality Index; HADS, Hospital Anxiety and Depression Scale.

**Table 2:** EORTC QLQ-C30: between-group analysis at 2 months after the beginning of the exercise (end of treatment) (T1).

EORTC QLQ-C30	T1 Group IG	T1 Group CG	*p*-Value
QL2	70.9 (25.0–100.0)	62.5 (41.7–66.7)	0.330
PF2	96.5 (86.7–100.0)	83.4 (60.0–87.0)	0.030 *
RF2	92.0 (50.0–100.0)	83.3 (83.0–84.0.)	0.228
EF	87.5 (58.0–100.0)	75.0 (58.3–75.0)	0.249
CF	100.0 (100.0–100.0)	74.8 (66.6–100.0)	0.018 *
SF	68.5 (50.0–100.0)	83.3 (50.0–100.0)	0.912
FA	22.8 (0.0–33.3)	55.6 (33.3–66.7)	0.017 *
NV	0.0 (0.0–16.6)	0.0 (0.0–0.0)	0.414
PA	0.0 (0.0–33.3)	8.4 (0.0–16.7)	0.807
DY	0.0 (0.0–0.0)	0.0 (0.0–0.0)	1000
SL	16.7 (0.0–66.7)	50.0 (0.0–66.7)	0.309
AP	0.0 (0.0–33.3)	0.0 (0.0–33.3)	0.759
CO	0.0 (0.0–66.7)	16.7 (0.0–33.3)	0.429
DI	0.0 (0.0–33.3)	0.0 (0.0–33.3)	0.789
FI	0.0 (0.0–0.0)	0.0 (0.0–33.3)	0.221

Abbreviations: IG, Intervention Group; CG, Control Group; EORTC QLQ-C30, European Organization for Research and Treatment of Cancer Quality of Life-C30 Questionnaire; QL, Global Health Status/Quality of Life; PF, Physical Functioning; RF, Role Functioning; EF, Emotional Functioning; CF, Cognitive Functioning; SF, Social Functioning; FA, Fatigue; NV, Nausea and Vomiting; PA, Pain; DY, Dyspnea; SL, Insomnia; AP, Appetite Loss; CO, Constipation; DI, Diarrhea; FI, Financial Difficulties. * *p*-value < 0.05.

**Table 3:** EORTC QLQ-C30: between-group analysis at 2 months from the end of the exercise training (T2).

EORTC QLQ-C30	T2 Group IG	T2 Group CG	*p*-Value
	(Median, Range)	(Median, Range)	
QL2	87.5 (75.0–91.7)	75.0 (66.7–83.3)	0.134
PF2	100.0 (93.3–100.0)	82.0 (73.3–87.0)	0.018 *
RF2	100.0 (100.0–100.0)	100.0 (100.0–100.0)	1.000
EF	95.8 (66.7–100.0)	91.7 (75.0–100.0)	0.762
CF	100.0 (100.0–100.0)	100.0 (100.0–100.0)	1.000
SF	91.7 (83.3–100.0)	92.5 (83.0–100.0)	1.000
FA	5.5 (0.0–11.0)	19.5 (11.0–22.2)	0.045 *
NV	0.0 (0.0–0.0)	0.0 (0.0–0.0)	1.000
PA	0.0 (0.0–33.3)	0.0 (0.0–16.7)	0.850
DY	0.0 (0.0–0.0)	0.0 (0.0–0.0)	1.000
SL	0.0 (0.0–33.3)	0.0 (0.0–33.3)	1.000
AP	0.0 (0.0–0.0)	0.0 (0.0–33.3)	0.317
CO	0.0 (0.0–33.3)	0.0 (0.0–33.3)	1.000
DI	0.0 (0.0–33.3)	0.0 (0.0–33.3)	1.000
FI	0.0 (0.0–0.0)	0.0 (0.0–33.3)	0.317

Abbreviations: IG, Intervention Group; CG, Control Group; EORTC European Organization for Research and Treatment of Cancer Quality of Life-C30 Questionnaire; QL, Global Health Status/Quality of Life; PF, Physical Functioning; RF, Role Functioning; EF, Emotional Functioning; CF, Cognitive Functioning; SF, Social Functioning; FA, Fatigue; NV, Nausea and Vomiting; PA, Pain; DY, Dyspnea; SL, Insomnia; AP, Appetite Loss; CO, Constipation; DI, Diarrhea; FI, Financial Difficulties. * *p*-value < 0.05.

**Table 4:** EORTC QLQ-C30: between-group analysis at 4 months from the end of the exercise training (T3).

EORTC QLQ-C30	T3 Group IG	T3 Group CG	*p*-Value
	(Median, Range)	(Median, Range)	
QL2	79.2 (50.0–100.0)	79.2 (75.0–100.0)	0.664
PF2	93.7 (60.0–100.0)	91.7 (87.0–100.0)	0.914
RF2	100.0 (80.0–100.0)	100.0 (100.0–100.0)	0.414
EF	87.9 (50.0–100.0)	89.4 (50.0–100.0)	0.914
CF	100.0 (100.0–100.0)	100.0 (100.0–100.0)	1.000
SF	100.0 (100.0–100.0)	100.0 (100.0–100.0)	1.000
FA	11.1 (0.0–44.0)	11.6 (0.0–33.0)	0.577
NV	0.0 (0.0–20.0)	0.0 (0.0–16.6)	0.693
PA	0.0 (0.0–33.3)	0.0 (0.0–23.3)	0.793
DY	0.0 (0.0–33.3)	0.0 (0.0–33.3)	0.759
SL	16.7 (0.0–66.0)	16.7 (0.0–66.7)	0.818
AP	0.0 (0.0–33.3)	0.0 (0.0–33.3)	0.789
CO	0.0 (0.0–33.3)	0.0 (0.0–33.3)	0.895
DI	0.0 (0.0–66.6)	0.0 (0.0–66.7)	0.648
FI	0.0 (0.0–0.0)	0.0 (0.0–0.0)	1.000

Abbreviations: IG, Intervention Group; CG, Control Group; EORTC QLQ-C30, European Organization for Research and Treatment of Cancer Quality of Life-C30 Questionnaire; QL, Global Health Status/Quality of Life; PF, Physical Functioning; RF, Role Functioning; EF, Emotional Functioning; CF, Cognitive Functioning; SF, Social Functioning; FA, Fatigue; NV, Nausea and Vomiting; PA, Pain; DY, Dyspnea; SL, Insomnia; AP, Appetite Loss; CO, Constipation; DI, Diarrhea; FI, Financial Difficulties.

**Table 5:** PSQI, HADS, Functional and Nutritional parameters: between-group analysis at 2 months after the beginning of the exercise (end of treatment) (T1), at 2 (T2), and 4 (T3) months from the end of the exercise training.

	Group IG	Group CG	*p*-Value
	(Median, Range)	(Median, Range)	
GLOBAL PSQI			
T0	8.5 (2.0–13.0)	7.0 (4.0–15.0)	0.855
T1	5.0 (3.0–10.0)	10.0 (4.0–12.0)	0.133
T2	3.0 (1.0–7.0)	4.0 (2.0–8.0)	0.554
T3	5.5 (3.0–10.0)	5.0 (2.0–9.0)	0.668
HADS: ANXIETY			
T0	7.0 (3.0–17.0)	6.0 (4.0–9.0)	0.783
T1	5.5 (0.0–14.0)	6.0 (2.0–8.0)	0.745
T2	4.0 (2.0–9.0)	5.0 (4.0–6.0)	0.554
T3	3.0 (1.0–9.0)	3.5 (0.0–8.0)	0.086
HADS: DEPRESSION			
T0	8.5 (2.0–14.0)	6.0 (4.0–8.0)	0.410
T1	4.5 (1.0–13.0)	5.5 (2.0–7.0)	0.830
T2	2.5 (0.0–16.0)	6.0 (3.0–7.0)	0.384
T3	2.0 (1.0–10.0)	3.0 (0.0–8.0)	0.104
SMWT			
T0	590.0 (360.0–685.0)	375.0 (330.0–560.0)	0.100
T1	625.0 (400.0–815.0)	359.0 (320.0–510.0)	0.022 *
T2	573.0 (445.0–815.0)	500.0 (400.0–535.0)	0.067
T3	580.0 (445.0–750.0)	450.0 (400.0–600.0)	0.100
SPPB			
T0	11.0 (7.0–12.0)	11.0 (9.0–11.0)	1.000
T1	11.0 (10.0–12.0)	11.0 (9.0–11.0)	0.161
T2	12.0 (10.0–12.0)	11.0 (10.0–12.0)	0.287
T3	12.0 (10.0–12.0)	10.0 (9.0–12.0)	0.169
HANDGRIP			
T0	57.0 (28.0–62.0)	41.0 (40.0–59.0)	0.272
T1	57.0 (28.0–62.0)	50.0 (41.0–55.0)	0.099
T2	57.0 (37.0–62.0)	52.0 (41.0–58.0)	0.233
T3	56.0 (39.0–65.0)	50.0 (41.0–55.0)	0.120
BMI			
T0	21.4 (17.2–25.7)	24.0 (22.0–41.4)	0.200
T1	21.8 (18.6–26.8)	24.0 (20.0–39.5)	0.465
T2	22.6 (19.5–27.6)	22.5 (20.0–39.5)	0.927
T3	23.0 (21.0–28.3)	22.5 (20.2–39.6)	0.647
SMI-BIA			
T0	8.9 (7.6–9.6)	7.8 (6.4–8.6)	0.086
T1	9.2 (7.6–10.5)	8.0 (6.5–10.7)	0.310
T2	9.7 (7.1–10.5)	8.0 (7.1–10.8)	0.582
T3	9.4 (7.0–9.7)	8.0 (7.4–10.9)	0.712
PhA-BIA			
T0	5.6 (4.1–5.7)	4.6 (4.5–5.6)	0.460
T1	5.6 (4.4–6.5)	5.0 (4.4–5.1)	0.141
T2	5.8 (5.0–6.5)	5.0 (4.4–5.3)	0.054
T3	5.8 (5.0–6.1)	5.0 (4.4–5.3)	0.022 *

Abbreviations: IG, Intervention Group; PSQI, Pittsburgh Sleep Quality Index; HADS, Hospital Anxiety and Depression Scale, 6MWT, 6 Minute Walking Test; SPPB, Short Physical Performance Battery; BMI, Body Mass Index; SMI-BIA, Skeletal Muscle Index Bio Impedance Analysis; PhA BIA, Phase Angle Bio Impedance Analysis. * *p*-value < 0.05.

**Table 6:** IG at baseline (T0), 2 months after the beginning of exercise (end of treatment) (T1), at 2 (T2), and 4 (T3) months from the end of the exercise training; *p*-value within IG.

IG (*n* = 6)	Follow-up					
	t0vst1	t0vst2	t0vst3	t1vst2	t1vst3	t2vst3
EORTC QLQ-C30	*p*-Value					
QL2	0.104	0.066	0.074	0.141	0.339	1.000
PF2	0.197	0.068	0.715	0.180	0.593	0.109
RF2	0.078	0.180	0.068	0.180	0.109	1.000
EF	0.116	0.068	0.075	0.655	0.893	1.000
CF	0.317	1.000	0.317	1.000	1.000	1.000
SF	0.078	0.068	0.027 *	0.180	0.068	0.157
FA	0.141	0.109	0.046 *	0.285	0.269	1.000
NV	0.285	1.000	1.000	0.317	0.414	0.317
PA	0.131	0.785	0.131	0.655	1.000	0.317
DY	0.317	1.000	1.000	1.000	0.317	1.000
SL	0.102	0.102	0.077	1.000	0.655	1.000
AP	0.141	0.317	0.221	0.317	0.564	0.157
CO	0.180	1.000	0.197	0.317	1.000	1.000
DI	0.564	0.317	0.705	0.317	1.000	0.317
FI	0.317	1.000	0.317	1.000	1.000	1.000
PSQI						
Global	0.078	0.144	0.339	0.180	0.752	0.357
HADS						
Anxiety	0.236	1.000	0.078	1.000	0.248	0.102
Depression	0.673	0.854	0.068	1.000	0.115	0.581
FUNCTIONAL PARAMETERS						
SMWT	0.028 *	0.173	0.075	0.465	0.172	0.892
HANDGRIP	0.715	0.917	0.600	0.285	0.500	0.686
SPPB	0.285	0.197	0.109	0.317	0.564	1.000
NUTRITIONAL PARAMETERS						
BMI	0.068	0.080	0.060	0.144	0.055	0.080
SMI-BIA	0.225	0.172	0.416	0.285	0.916	0.167
PhA-BIA	0.109	0.028 *	0.027 *	0.109	0.206	1.000

Abbreviations: IG, Intervention Group; EORTC QLQ-C30, European Organization for Research and Treatment of Cancer Quality of Life-C30 Questionnaire; QL, Global Health Status/Quality of Life; PF, Physical Functioning; RF, Role Functioning; EF, Emotional Functioning; CF, Cognitive Functioning; SF, Social Functioning; FA, Fatigue; NV, Nausea and Vomiting; PA, Pain; DY, Dyspnea; SL, Insomnia; AP, Appetite Loss; CO, Constipation; DI, Diarrhea; FI, Financial Difficulties; PSQI, Pittsburgh Sleep Quality Index; HADS, Hospital Anxiety and Depression Scale, 6MWT, 6 Minute Walking Test; SPPB, Short Physical Performance Battery; BMI, Body Mass Index; SMI-BIA, Skeletal Muscle Index Bio Impedance Analysis; Pha BIA, Phase Angle Bio Impedance Analysis; * *p*-value < 0.05. The underline indicates the various outcome measures.

**Table 7:** CG at baseline (T0), 2 months after the beginning of exercise (end of treatment) (T1), at 2 (T2) and 4 (T3) months from the end of the exercise training; *p*-value within CG.

CG (*n* = 5)	Follow-up					
	t0vst1	t0vst2	t0vst3	t1vst2	t1vst3	t2vst3
EORTC QLQ C30	*p*-Value					
QL2	0.715	1.000	0.066	0.141	0.066	0.109
PF2	0.102	1.000	0.180	0.655	0.109	0.109
RF2	0.357	1.000	0.109	0.066	0.066	1.000
EF	1.000	0.066	0.715	0.109	0.144	0.715
CF	0.276	0.066	0.066	0.102	0.102	1.000
SF	0.285	0.285	0.180	0.285	0.180	0.180
FA	0.180	0.144	0.144	0.068	0.068	0.715
NV	0.317	0.317	0.655	1.000	0.317	0.317
PA	0.593	0.276	0.180	0.276	1.000	0.655
DY	0.317	0.317	1.000	1.000	0.317	0.317
SL	0.655	0.141	0.180	0.276	0.317	0.655
AP	0.317	1.000	0.317	0.655	1.000	0.655
CO	0.317	0.655	1.000	0.655	0.564	0.655
DI	0.317	0.317	0.317	0.317	0.655	0.655
FI	1.000	0.317	0.317	0.317	0.317	0.317
PSQI						
Global	1.000	0.066	0.066	0.066	0.066	0.785
HADS						
Anxiety	0.581	0.141	0.197	1000	0.109	0.785
Depression	0.715	0.465	0.285	0.715	0.197	0.854
FUNCTIONAL PARAMETERS						
SMWT	0.225	0.223	0.860	0.138	0.155	0.715
HANDGRIP	0.465	0.465	0.465	0.465	0.083	0.705
SPPB	1.000	0.414	1.000	0.414	1.000	0.414
NUTRITIONAL PARAMETERS						
BMI	0.144	0.078	0.078	1.000	0.713	0.063
SMI-BIA	0.176	0.176	0.131	0.109	0.109	0.593
PHA-BIA	0.786	0.588	0.498	0.180	0.180	1.000

Abbreviations: CG, Control Group; EORTC, European Organization for Research and Treatment of Cancer Quality of Life-C30 Questionnaire; QL, Global Health Status/Quality of Life; PF, Physical Functioning; RF, Role Functioning; EF, Emotional Functioning; CF, Cognitive Functioning; SF, Social Functioning; FA,6 Fatigue; NV, Nausea and Vomiting; PA, Pain; DY, Dyspnea; SL, Insomnia; AP, Appetite Loss; CO, Constipation; DI, Diarrhea; FI, Financial Difficulties; PSQI, Pittsburgh Sleep Quality Index; HADS, Hospital Anxiety and Depression Scale, 6MWT, 6 Minute Walking Test; SPPB, Short Physical Performance Battery; BMI, Body Mass Index; SMI-BIA, Skeletal Muscle Index Bio Impedance Analysis; Pha BIA, Phase Angle Bio Impedance Analysis; The underline indicates the various outcome measures.

## Data Availability

The data presented in this study are available on request from the corresponding author.
